# Spotlighting Disability in a Major Electronic Health Record: Michigan Medicine’s Disability and Accommodations Tab

**DOI:** 10.2196/38003

**Published:** 2022-12-02

**Authors:** Heather Halkides, Tyler G James, Michael M McKee, Michelle A Meade, Christa Moran, Sophia Park

**Affiliations:** 1 Health Information & Technology Services Michigan Medicine Ann Arbor, MI United States; 2 Department of Family Medicine University of Michigan Ann Arbor, MI United States; 3 Center for Disability Health and Wellness Michigan Medicine University of Michigan Medical School Ann Arbor, MI United States; 4 Department of Physical Medicine and Rehabilitation University of Michigan Ann Arbor, MI United States; 5 Interpreter Services Office of Patient Experience Michigan Medicine Ann Arbor, MI United States

**Keywords:** patients with disabilities, disability accommodations, electronic health records, patient-centered care, Affordable Care Act, Americans with Disabilities Act, disability, disabilities, affordable care, EHR, accommodation, minority, equity, accessibility, accessible, inclusive, inclusivity, health care, health service, environment, accommodate, reporting, data collection, barrier

## Abstract

People with disabilities represent the largest minority group in the United States and a priority population for health services research. Despite federal civil rights law, people with disabilities face inaccessible health care environments that fail to accommodate their disability. We present Michigan Medicine’s Disability and Accommodations Tab. This patient-facing questionnaire and shared data field in the electronic health record enables the collection and reporting of patient disability-related accommodations. The Disability Tab seeks to address provider- and clinic staff–reported barriers to providing accommodations and fosters an opportunity to redesign health care to meet the needs of people with disabilities.

## Introduction

People with disabilities represent a considerable proportion of the US population, with 27% of adults (in 2019) and 4% of children (in 2019) having a disability [1,2]. This population experiences widespread health inequities, largely due to stigma in society and inaccessibility of health care services. Due to these inequities, national public health and health care organizations (eg, the Agency for Health care Research and Quality [3]) deem people with disabilities as a priority population in health services research and detail several national health objectives (eg, Healthy People 2030 [4]) to improve the health of this population. To achieve these health objectives, however, we must address the inaccessibility of health care services and tailor these services to meet the needs of people with disabilities [5].

Federal civil rights laws in the United States—including Section 504 of the Rehabilitation Act of 1973, the Americans with Disabilities Act of 1990, and Section 1557 of the Patient Protection and Affordable Care Act—delineate the responsibility of health care organizations to be accessible to people with disabilities. Despite these mandates, many health care environments remain inaccessible to people with disabilities [6-13]. The failure to provide disability accommodations appears, at least in part, due to a lack of knowledge on disability accommodations among care team members. A recent study by Iezzoni et al [14] found that 71% of US physicians provided incorrect answers on who makes decisions about reasonable accommodations for people with disabilities, and 68% believed they were at risk for an Americans with Disabilities Act lawsuit because of accommodation issues. Misunderstanding accommodation needs and responsibilities may be due to the fact that disability accommodations are not systematically addressed in most health care systems. In many health systems, people with disabilities who require disability-related accommodations have to request the accommodation to care team staff prior to or during every medical encounter [7,15]. A lack of advanced knowledge of accommodation needs is a primary barrier to providing accommodations that can be attributed to a lack of widescale, systematic accommodation reporting [15]. This lack of centralized reporting of patient disability status and requested accommodations has been a source of inaccessibility for people with disabilities and an increasing risk for litigation for health systems [15-17].

To address barriers in communicating accommodations, the Centers for Medicare and Medicaid Services recommends collecting disability-related information at the point of care [18]. The use of health informatics, specifically electronic health records (EHRs), can improve the provision of accommodations for people with disabilities, and therefore, improve the delivery of patient care and promote health equity [15,17,19,20]. However, the description of tools to systematically collect patient disability-related information is limited.

## Michigan Medicine’s Disability and Accommodations Tab

### Development

Michigan Medicine, the health care system owned and operated by the University of Michigan Medical School, is one of the largest health care systems in Michigan, serving over 2.7 million patient encounters per year at 3 hospitals and 40 outpatient clinics. Michigan Medicine has already developed strong commitments to improving the health of people with disabilities, including the establishment of model clinics, such as the Deaf Health Clinic in Family Medicine, and recognition and financial support for the Center for Disability Health and Wellness [21,22].

**Figure 1:**
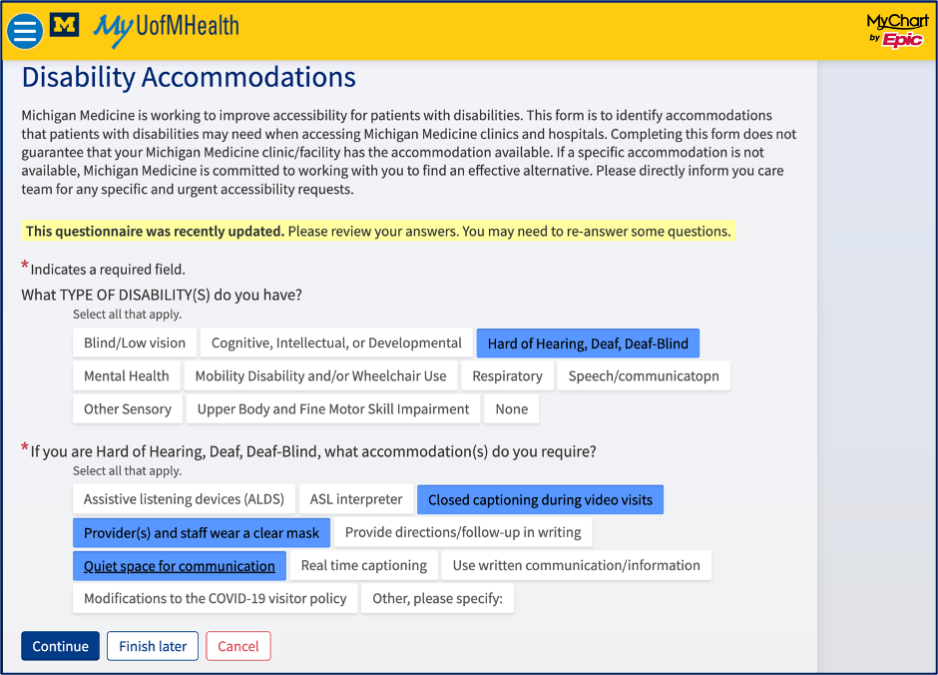
Example of the Disability and Accommodations Tab questionnaire in the patient-facing portal. As the tool is specific to requesting disability-related accommodations, the requested accommodation is required after selecting a disability classification. © 2022 Epic Systems Corporation. The MyUofMHealth App is powered by MyChart® licensed from Epic Systems Corporation, © 1999-2022.

In 2019, Michigan Medicine’s faculty and staff (including CM and MMM), with the support of Michigan Medicine’s Disability Resource Group, met with MiChart Ambulatory Team (including HH) to create the Disability and Accommodations Tab (or “Disability Tab”). The Disability Tab is a shared data field based on a questionnaire (ie, SmartForm) within Michigan Medicine’s version of Epic, called MiChart, which collects discrete data from both the patient-facing portal (Figure 1) and care team members. Patients wishing to report needed disability-related accommodations through the patient portal can complete the optional questionnaire in the same location as the Gender Identity, Sexual Orientation, and Poke Plan questionnaires. These questionnaires have the functionality to be routinely pushed to patients prior to ambulatory care encounters for intake. For care team members, the Disability Tab questionnaire can be completed through MiChart. The description of the questionnaire in the patient portal is as follows:

Michigan Medicine is working to improve accessibility for patients with disabilities. This form is to identify accommodations that patients with disabilities may need when accessing Michigan Medicine clinics and hospitals. Completing this form does not guarantee that your Michigan Medicine clinic or facility has the accommodation available. If a specific accommodation is not available, Michigan Medicine is committed to working with you to find an effective alternative. Please directly inform you care team for any specific and urgent accessibility requests.

Initial disability classifications and accommodation options listed in the Disability Tab were created by subject matter experts at Michigan Medicine in collaboration with people with disabilities, disability advocacy groups and service centers, and care team staff who work routinely with people with disabilities. In June of 2021, the University of Michigan’s Center for Disability Health and Wellness established a work group to manage the development, pilot testing, and implementation of the Disability Tab. This group further refined the disability and accommodation options to meet federal, state, and local regulatory guidance, in addition to common accommodations for different disabilities (Table 1). For example, initially we implemented a general accommodation to indicate the need for visitor or mask exemptions related to the COVID-19 pandemic. Based on guidance from the Centers for Disease Control and Prevention [23] and Michigan Medicine’s Patient Civil Rights Coordinator, we refined the accommodations to be disability-specific (eg, a patient with blindness or low vision may have an indicated need for a visitor to be present to assist but not a mask mandate exemption).

Of note, the disability options listed on the Disability Tab differ from the Washington Group questions and the American Community Survey questions, as those specific questions measure only functional or activity limitations (eg, difficulty seeing and difficulty concentrating) [24,25]. For instance, one question on the American Community Survey asks, *“*Because of a physical, mental, or emotional problem, do you have difficulty doing errands alone such as visiting a doctor’s office or shopping?” This question combines multiple functionally and qualitatively different disability categories that have different indicated accommodation needs. To assist in streamlining the provision of accommodations, we opted to be specific with respect to the type of disability a patient presents.

**Figure 2:**
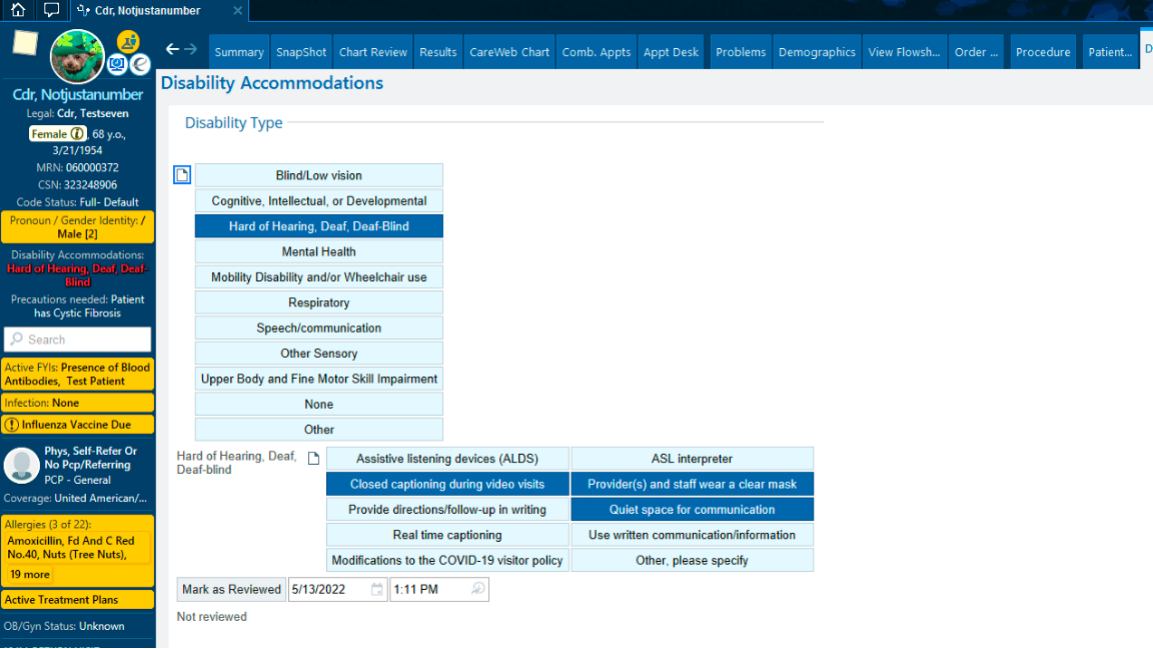
Example of the Disability and Accommodations Tab on the patient Storyboard in MiChart. © 2022 Epic Systems Corporation.

Data from this questionnaire are displayed on patient’s Storyboard in MiChart (Figure 2), notifying care team members of the patient’s disability and accommodation needs. On the Storyboard, in the patient’s demographic section, there is a section for disability accommodations displayed next to medical interpreter needs and gender identity. The brief section in the demographic column identifies only the patient’s indicated disability or disabilities. MiChart users can then click this section to open a section of the medical record listing the indicated accommodation needs.

The Disability Tab became active in the EHR and patient portal in October 2020. As of December 2021, however, the Disability Tab had not been promoted by Michigan Medicine nor had systematic data collection been incorporated into the clinic workflows due to the COVID-19 pandemic. In addition, no patient portal (MyChart or MyUofMHealth) reminder had prompted patients to complete the questionnaire. Despite this lack of promotion, as of December 13, 2021, almost 3000 patients (n=2941) had completed questionnaires (for reference, Michigan Medicine has over 240,000 active primary care patients). Among these patients, 1 in 4 (n=738, 25.1%) report mobility disabilities, followed by patients reporting mental health disabilities (n=441, 15%); patients who are deaf, hard of hearing, or deafblind (n=426, 14.5%); patients with cognitive disabilities (n=388, 13.2%); speech or other communication disabilities (n=209, 7.1%); blindness or low vision (n=185, 6.3%); and upper body or fine motor skill impairment (n=161, 5.5%).

Several health system wide initiatives have recognized the potential benefits of the Disability Tab to collect and display information about accommodation needs. One of these initiatives was the May 2022 rollout of MyChart Bedside, a tablet-based inpatient portal tool to improve the patient experience. The use of MyChart Bedside enables patients to complete the Disability Tab questionnaire during their inpatient stays directly from their MyChart Bedside tablets. These patient responses populate the Disability Tab field in the Storyboard for care team members to see. As of September 1, 2022, a total of 4732 patients have Disability Tab information in their medical records. Among these patients, mobility disabilities and wheelchair use were the most common (n=1134, 24%), followed by no disabilities (n=939, 19.8%); ‘other’ disabilities (n=800, 16.9%); hard of hearing, deafness, or deafblindness (n=793, 16.8%); mental health disabilities (n=676, 14.3%); cognitive disabilities (n=653, 13.8%); speech disabilities (n=356, 7.5%); blindness (n=330, 7%), upper body and fine motor skill impairment (n=256, 5.3%); other sensory disabilities (n=223, 4.7%); and respiratory disabilities (n=43, 0.9%).

**Table 1:** Disability and accommodation options in Michigan Medicine’s Disability and Accommodations Tab.

Disability classification	Available accommodation options
Blind/low vision	Audio descriptors Braille Provide documents in large print Screen readers Human guide Exceptions to the COVID-19 visitor policy Other, please specify
Cognitive, intellectual, or developmental	Assistance with completing surveys/patient intake Check for understanding Closed captioning during video visits I want to give people information in advance, before going to the clinic (see ‘other’) I have a support person, please involve them in my medical discussions Provide directions/follow-up in writing Use visuals or pictures to explain concepts Modifications to the COVID-19 mask policy Modifications to the COVID-19 visitor policy Need for reduced sensory input Other, please specify
Hard of hearing, deaf, or deafblind	Assistive listening devices ASL^a^ interpreter Closed captioning during video visits Provider(s) and staff wear a clear mask Provide directions/follow-up in writing Quiet space for communication Real-time captioning Use written communication or information Modifications to the COVID-19 visitor policy Other, please specify
Mental health	Additional structure and assistance regulating emotions Clear protocols to help me prepare for care Need for reduced sensory input Provide directions/follow-up in writing Modifications to the COVID-19 mask policy Modifications to the COVID-19 visitor policy Other, please specify
Mobility disability or wheelchair use	Adjustable tables Assistance with transfers and walking Availability of transfer equipment (eg, a lift, a transfer board) Human assistance with transfers Larger exam rooms Wheelchair scales Modifications to the COVID-19 mask policy Modifications to the COVID-19 visitor policy Other, please specify
Respiratory	Modifications to the COVID-19 mask policy Need for oxygen tank Plug outlet for oxygen concentrator Other, please specify
Speech/communication	Closed captioning during video visits Confirm that I understand Give me additional time to speak Understanding prompts from the provider Whiteboards for communication Other, please specify
Other sensory	Fragrance-free environment Limit touch Placement in room early Other, please specify
Upper body and fine motor skill impairment	Assistance with clothing management Assistance with completing surveys/patient intake Assistance with transfers Modifications to the COVID-19 mask policy Modifications to the COVID-19 visitor policy Other, please specify
Other (please specify)	Other, please specify
None	—^b^

^a^ASL: American Sign Language.

^b^—: Not applicable.

### Updating the Disability Tab

Modifications to the SmartForm used for the Disability Tab requires substantial time for revising the code and testing for quality assurance before revisions are ‘live’ in the EHR. Further, ‘retiring’ disability or accommodation categories can lead to missing data if not properly coded. For this reason, we have implemented a Change Review Board process to review requested changes (eg, adding an accommodation field) with respect to the financial, reporting, and human resource impacts. The Disability Tab Change Review Board solicits regulatory feedback from both the Americans with Disabilities Act Coordinator and Patient Civil Rights Coordinator, and other interested parties (eg, the Office of Patient Experience).

With the implementation of the Disability Tab in MyChart Bedside (in May 2022), we recognized the need for a ‘mark as reviewed’ function on the questionnaire. This is particularly important when patient disability-related accommodation needs change due to onset or progression of disability. To address this need, we developed a button on the SmartForm that records the last user and the date or time the questionnaire was reviewed.

### Identifying Opportunities for Ambulatory Care Workflow Integration

To identify opportunities to integrate disability data collection within the workflow in outpatient clinics, the Disability Tab started being actively used (eg, patients’ disability-related needs being documented by the care team on the Disability Tab) at the Dexter Health Center, operated by Michigan Medicine’s Department of Family Medicine, in March 2022. At Dexter Health Center, patients are asked to complete a paper version of the Disability Tab questionnaire, which then gets added to the electronic Disability Tab by clerical or medical assistants. Once entered, this information becomes available across all clinical encounters across Michigan Medicine Health Systems. Staff at this clinic are using the Disability Tab to prepare accommodations in advance of future clinic appointments. To understand modifications to their workflow and acceptability among care team staff, we are collecting data in an ongoing quality improvement study. Results of this study, focused on operation workflow of the Disability Tab (for collecting information and providing accommodations), will be disseminated at a later date.

## Discussion

### Principal Findings

With the widespread lack of centralized disability services in health care systems, health care providers and clinic staff are often responsible for determining and providing disability accommodation needs [16]. This process, however, presents challenges, particularly with staff communication across patient encounters regarding disability accommodations. Studies, including an unpublished quality improvement study at Michigan Medicine [26], find that being unaware of the need for an accommodation in advance is a common barrier to providing accommodations to people with disabilities [15]. This is concerning since clinic staff are integral to arranging accommodations for upcoming appointments for people with disabilities.

The Disability Tab provides an opportunity to specifically address provider and clinic staff barriers in providing accommodations to people with disabilities. Patients can report their specific disabilities and accommodation needs by filling out the questionnaire within the patient portal or being prompted by care team staff at the point of care; this information appears for health care providers and clinic staff in the Storyboard, as part of the patient’s demographic information. Moreover, this tool provides the opportunity to identify and articulate the role of caregivers as well as any alterations related to patient autonomy, addressing barriers often experienced by patients who have cognitive, intellectual, or developmental disabilities.

By design, the Disability Tab reports disability-related accommodation needs information systemwide, making this available to all care team members across all clinical encounters, not just the patients’ primary care providers, or hidden within free-text clinical notes, as is common in health care [15]. This tool is critical in minimizing the information gap during patient transitions and handoffs. Moreover, the development of the Disability Tab in Epic, a major EHR system, allows for significant scalability to other health care systems, as this field could be requested and implemented by other systems that use Epic. As such, the design and implementation of the Disability Tab has the potential to facilitate the identification of individuals with disability and the standardization of disability-related information in health care as outlined under the Section 4302 of the Affordable Care Act [18].

### Limitations and Future Work

The Disability Tab is still relatively early in the implementation phase and there are several opportunities for future work, particularly to respond to potential limitations. First, the Disability Tab questionnaire uses disability categories that are not standardized to the American Community Survey or Washington Group disability items. Therefore, information from the Disability Tab will not facilitate population health comparisons. Although this may be a limitation, we determined early in the Disability Tab’s development that the goal of this tool was to facilitate accommodation access, not solely collect disability prevalence information [17,20].

Given the early stage of implementation, there are several outstanding questions. one question is whether the presence of disability identity impacts the process of care. For example, during the initial impact of the COVID-19 pandemic, there was considerable discussion of rationing (ie, declining) care to people with disabilities [27,28]. How care team members use the information for care, outside of simply providing accommodations, is undetermined. An additional concern is how to clearly establish a workflow that enables outpatient clinics and inpatient services to provide requested accommodations. The Disability Tab meets the need of providing information to care team staff; it does not, however, ensure that staff are interacting with or acting on this information. Lastly, as with all clinical informatics interventions, there are concerns that the Disability Tab may further widen the gap among people with disabilities who are multiply marginalized through intervention-generated inequality [29]. The Disability Tab is currently only available in English, and it is possible that patients with limited English proficiency are not asked their disability and accommodation needs at the bedside. Further, patients who do not have reliable internet access or patient portal access are unable to complete the questionnaire online. Future efforts should focus on further equitizing this intervention.

### Conclusions

In its infancy, the Disability Tab demonstrates the opportunity to leverage EHR systems and health informatics to systematically collect disability-related accommodation needs to improve the quality of care delivered to people with disabilities and improve accessibility to health care environments. We encourage other health care systems to adopt similar approaches to address the health care needs of people with disabilities.

## Abbreviations

EHR: electronic health record
